# Different Anti-Contractile Function and Nitric Oxide Production of Thoracic and Abdominal Perivascular Adipose Tissues

**DOI:** 10.3389/fphys.2016.00295

**Published:** 2016-07-12

**Authors:** Jamaira A. Victorio, Milene T. Fontes, Luciana V. Rossoni, Ana P. Davel

**Affiliations:** ^1^Department of Structural and Functional Biology, Institute of Biology, University of CampinasCampinas, Brazil; ^2^Vascular Physiology Lab, Department of Physiology and Biophysics, Institute of Biomedical Sciences, University of São PauloSão Paulo, Brazil

**Keywords:** thoracic aorta, abdominal aorta, nitric oxide, oxidative stress, perivascular adipose tissue, endothelium

## Abstract

Divergent phenotypes between the perivascular adipose tissue (PVAT) surrounding the abdominal and the thoracic aorta might be implicated in regional aortic differences, such as susceptibility to atherosclerosis. Although PVAT of the thoracic aorta exhibits anti-contractile function, the role of PVAT in the regulation of the vascular tone of the abdominal aorta is not well defined. In the present study, we compared the anti-contractile function, nitric oxide (NO) availability, and reactive oxygen species (ROS) formation in PVAT and vessel walls of abdominal and thoracic aorta. Abdominal and thoracic aortic tissue from male Wistar rats were used to perform functional and molecular experiments. PVAT reduced the contraction evoked by phenylephrine in the absence and presence of endothelium in the thoracic aorta, whereas this anti-contractile effect was not observed in the abdominal aorta. Abdominal PVAT exhibited a reduction in endothelial NO synthase (eNOS) expression compared with thoracic PVAT, without differences in eNOS expression in the vessel walls. In agreement with this result, NO production evaluated *in situ* using 4,5-diaminofluorescein was less pronounced in abdominal compared with thoracic aortic PVAT, whereas no significant difference was observed for endothelial NO production. Moreover, NOS inhibition with L-NAME enhanced the phenylephrine-induced contraction in endothelial-denuded rings with PVAT from thoracic but not abdominal aorta. ROS formation and lipid peroxidation products evaluated through the quantification of hydroethidine fluorescence and 4-hydroxynonenal adducts, respectively, were similar between PVAT and vessel walls from the abdominal and thoracic aorta. Extracellular superoxide dismutase (SOD) expression was similar between the vessel walls and PVAT of the abdominal and thoracic aorta. However, Mn-SOD levels were reduced, while CuZn-SOD levels were increased in abdominal PVAT compared with thoracic aortic PVAT. In conclusion, our results demonstrate that the anti-contractile function of PVAT is lost in the abdominal portion of the aorta through a reduction in eNOS-derived NO production compared with the thoracic aorta. Although relative SOD isoforms are different along the aorta, ROS formation, and lipid peroxidation seem to be similar. These findings highlight the specific regional roles of PVAT depots in the control of vascular function that can drive differences in susceptibility to vascular injury.

## Introduction

Aortic atherosclerotic lesion and aneurysm are predominant in the abdominal rather than the thoracic portion of the aorta, suggesting inherent properties influencing regional aortic susceptibility to injury; however, the mechanisms involved in this susceptibility are not well understood. Despite being segments of the same artery, elastic features, and collagen contents vary along the aorta, which contribute to the physiological characteristics of pulse wave and blood distribution (Sokolis et al., 2008; Tsamis et al., 2013). Moreover, although the levels of endothelium-dependent relaxation induced by acetylcholine appear to be similar (Oloyo et al., 2012), the mechanisms involved in the alpha-adrenergic-mediated contraction might differ in the thoracic vs. the abdominal aorta (Lamb et al., 1994; Asbún-Bojalil et al., 2002).

It has been demonstrated that perivascular adipose tissue (PVAT), an adipose depot surrounding most arteries, plays an important role in vascular homeostasis (Szasz and Webb, 2012). PVAT of murine thoracic aorta secretes vasoactive substances, including adiponectin (Fésus et al., 2007), angiotensin 1–7 (Lee et al., 2009), leptin (Gálvez-Prieto et al., 2012), H_2_S (Fang et al., 2009), and a still unidentified adipocyte-derived relaxing factor(s) (ADRFs; Fésus et al., 2007; Schleifenbaum et al., 2010; Oriowo, 2015). These vasoactive factors can induce an anti-contractile effect through endothelial nitric oxide (NO) synthesis and release (Gálvez-Prieto et al., 2012) and/or through activation of vascular smooth muscle K^+^ channels (Gao et al., 2007). More recently, it was also demonstrated that PVAT from the thoracic aorta expresses the endothelial isoform of NO synthase (eNOS; Araujo et al., 2015; Xia et al., 2016) and produces NO. PVAT-derived NO mediates relaxation of the adjacent thoracic aortic wall, indicating NO as a potential ADRF in this vessel (Xia et al., 2016).

Along the aorta, the phenotype of the PVAT is distinguished. Abdominal aorta PVAT resembles the phenotype of white adipose tissue (WAT), while fat surrounding the thoracic aorta shares the characteristics of brown adipose tissue (BAT; Police et al., 2009; Fitzgibbons et al., 2011). In addition, the expression levels of inflammatory genes and markers of immune cell infiltration are greater in abdominal PVAT than in thoracic PVAT, suggesting that the WAT phenotype is more pro-inflammatory and atherogenic than the BAT phenotype in PVAT (Padilla et al., 2013). Accordingly, PVAT from human coronary arteries with the WAT phenotype exhibits a pro-inflammatory profile with reduced adiponectin levels compared to BAT subcutaneous and peri-renal adipocyte depots (Chatterjee et al., 2009).

Although the anti-contractile and anti-inflammatory effects of thoracic aortic PVAT are well-known, the possible role of PVAT regulating the vascular reactivity and redox status of the abdominal aorta is still poorly understood. A previous study demonstrated that the anti-contractile effect of abdominal aortic PVAT is less pronounced when compared to the thoracic portion of the aorta (Watts et al., 2011). However, the mechanism involved in this regional difference remains unclear. This study aimed at investigating the comparative effects of thoracic vs. abdominal aortic PVAT on anti-contractile function, the oxidative profile, and NO synthesis and availability.

## Methods

### Animals

All animal procedures were in accordance with the ethical principles for animal experimentation adopted by the Brazilian Society of Laboratory Animal Science (SBCAL/COBEA) and were approved by the Ethics Committee on Animal Use of the University of Campinas—UNICAMP (protocol number: 3523-1) and of the Institute of Biomedical Science at the University of Sao Paulo (protocol number 53, sheet 19, book 03).

Experiments were conducted in 3- to 4-month-old male Wistar rats (Multidisciplinary Center for Biological Research, UNICAMP) maintained at a constant room temperature (22–24⋅C) and light cycle (12:12 h light:dark) with food and water allowed *ad libitum* to all animals. At the time of the experiments, animals were euthanized under anesthesia (ketamine 80 mg/kg and xylazine 5 mg/kg; *i.p*.).

### Vascular reactivity study

Thoracic and abdominal aorta were isolated, placed in Petri dishes with cold Krebs-Henseleit solution (in mM: 118 NaCl, 4.7 KCl, 25 NaHCO_3_, 2.5 CaCl_2_-2H_2_O, 1.2 KH_2_PO_4_, 1.2 MgSO_4_-7H_2_O, 11 glucose and 0.01 EDTA) and sectioned into 3-mm rings with or without PVAT. In some experiments, endothelial cells were removed by gently rolling the preparation back and forth with a needle. Rings were mounted at a resting tension of 1 g in an organ chamber bath (Panlab Harvard Apparatus, Cornellà - Barcelona, Spain) containing Krebs–Henseleit solution continuously aerated with 95% O_2_, 5% CO_2_ (pH = 7.4; 37⋅C) as previously described (Davel et al., 2006, 2008). Isometric tension was recorded using an isometric force transducer (MLT0420, AdInstruments) connected to an acquisition PowerLab 8/30 system for tension recording (LabChart 7, AdInstruments).

After a 30-min equilibration period, all aortic rings were initially exposed twice to 75 mM KCl, the first to check their functional integrity and the second to assess the maximum contractility. Following the wash, vascular reactivity was investigated with cumulative concentration-response curves to acetylcholine (0.1 nM – 10 μM) in vessels contracted with phenylephrine (1 μM). Endothelial denudation was confirmed by acetylcholine-induced relaxation < 10%. After the samples were washed, the contractile response to phenylephrine (1 nM – 10 μM for thoracic aorta and 1 nM – 100 μM for abdominal aorta) was evaluated. Some aortic rings with PVAT and without endothelium were incubated with NO synthase inhibitor L-NAME (100 μM) for 30 min prior to phenylephrine concentration-response curves.

At the end of the experiments, the length of each ring was measured. Vasoconstrictor responses to phenylephrine are expressed as mN/mm. Relaxation responses to acetylcholine are expressed as the percentage of relaxation of the contractile response induced by phenylephrine (1 μM).

### Nitric oxide (NO) and reactive oxygen species (ROS) evaluation *in situ*

Thoracic and abdominal aortic tissues with their respective coated PVAT were isolated, placed in Petri dishes with cold Krebs-Henseleit solution, cleaned and sectioned into 3-mm rings. Next, in a dark chamber, aortic segments were incubated for 30 min with Krebs-Henseleit solution (pH = 7.4, 37⋅C) plus 4,5-diaminofluorescein diacetate (DAF-2, 10 μM) or with dihydroethidium (DHE, 2 μM) for NO and ROS measurement, respectively, as previously described (Gil-Ortega et al., 2014). Some aortic segments were co-incubated with DAF-2 plus L-NAME (1 mM) to evaluate the specific generation of NO from NO synthase or with DHE plus MnTMPyP (25 μM), a cell-permeant superoxide dismutase (SOD) mimetic, to evaluate superoxide anion formation. Subsequently, the aortic segments with PVAT were fixed in 4% paraformaldehyde for 4 h and then embedded in freezing medium (Tissue-Tek, Sakura Finetek, Torrance, CA). Transverse sections (20 μm thick) of frozen arteries were obtained on a cryostat. Digital images were collected on a microscope (Nikon, Chiyoda-ku, Tokyo, Japan) equipped with epifluorescence and fluorescein/rhodamine filters using a 20X objective. The images were analyzed using ImageJ software (NIH, Bethesda, MD, USA). NO availability was evaluated by DAF-2 mean optical density of the fluorescence in the endothelium and PVAT, and ROS production were analyzed based on the integrated density of the DHE fluorescence normalized by the number of nuclei labeled with ethidium bromide (EB-positive nuclei) in the vascular wall segment of the aorta and its respective PVAT.

### Western blotting

Total protein extracts were obtained from abdominal and thoracic aortic samples and their respective PVAT depots. Tissues were homogenized in cold RIPA lysis buffer (Merck Millipore, Billerica, MA, USA) containing phenylmethylsulfonyl fluoride (1 mM PMSF), Na_3_VO_4_ (1 mM) and protease inhibitor cocktail (2 μL/mL PIC, Sigma-Aldrich).

Protein extracts (75 μg) were separated by SDS–PAGE, and proteins were transferred to PVDF membranes (GE HealthCare, Little Chalfont-Buckinghamshire, UK) using a Mini Trans-Blot Cell system (Bio-Rad, Hercules, CA, USA) containing 25 mM Tris, 190 mM glycine, 20% methanol and 0.05% SDS. Membranes were blocked for 90 min at room temperature with 5% albumin in Tris buffer (10 mM Tris, 100 mM NaCl and 0.1% Tween 20). Membranes were then incubated overnight at 4⋅C with the primary antibodies anti-eNOS (1:750; BD Transduction, Franklin Lakes, NJ, USA), anti-EC-SOD (1:1,000; Enzo Life Science, Farmingdale, New York, USA), anti-Mn-SOD (1:1,000; Enzo Life Science), anti-CuZn-SOD (1:5,000; Sigma Aldrich), and anti-4-hydroxynonenal (4-HNE; 1:2,000; Abcam, Cambridge, UK).

After washing, membranes were incubated for 90 min with a peroxidase-conjugated IgG antibody specific for the primary antibody used. Protein expression was detected with Pierce ECL Western Blotting Substrate (Thermo Scientific, Rockford, IL, USA), and the membranes were subjected to autoradiography (Amersham Hyperfilm ECL, GE Healthcare). The blots were digitized, and intensity was quantified using ImageJ 1.46p software (National Institutes of Health). Ponceau staining was used to normalize expression of the evaluated proteins in each sample.

### Drugs

Acetylcholine chloride, phenylephrine hydrochloride, L-NAME, and DAF-2 were purchased from Sigma-Aldrich CO (Saint Louis, MO, USA). MnTMPyP was purchased from Calbiochem (Merck Millipore). DHE was purchased from Invitrogen (Grand Island, NY, USA).

### Statistical analysis

Results are expressed as the means ± SEM. Data were analyzed using GraphPad Prism 5.0 software (GraphPad Software Corp., USA). Concentration–response curves were analyzed using two-way ANOVA followed by the Bonferroni's post-test. For comparisons between abdominal and thoracic aortic samples in the same condition, the Student *t*-test was used. Values of *P* < 0.05 were considered significantly different.

## Results

### PVAT exerts an anti-contractile effect in the thoracic but not the abdominal aorta

**Figure 1:**
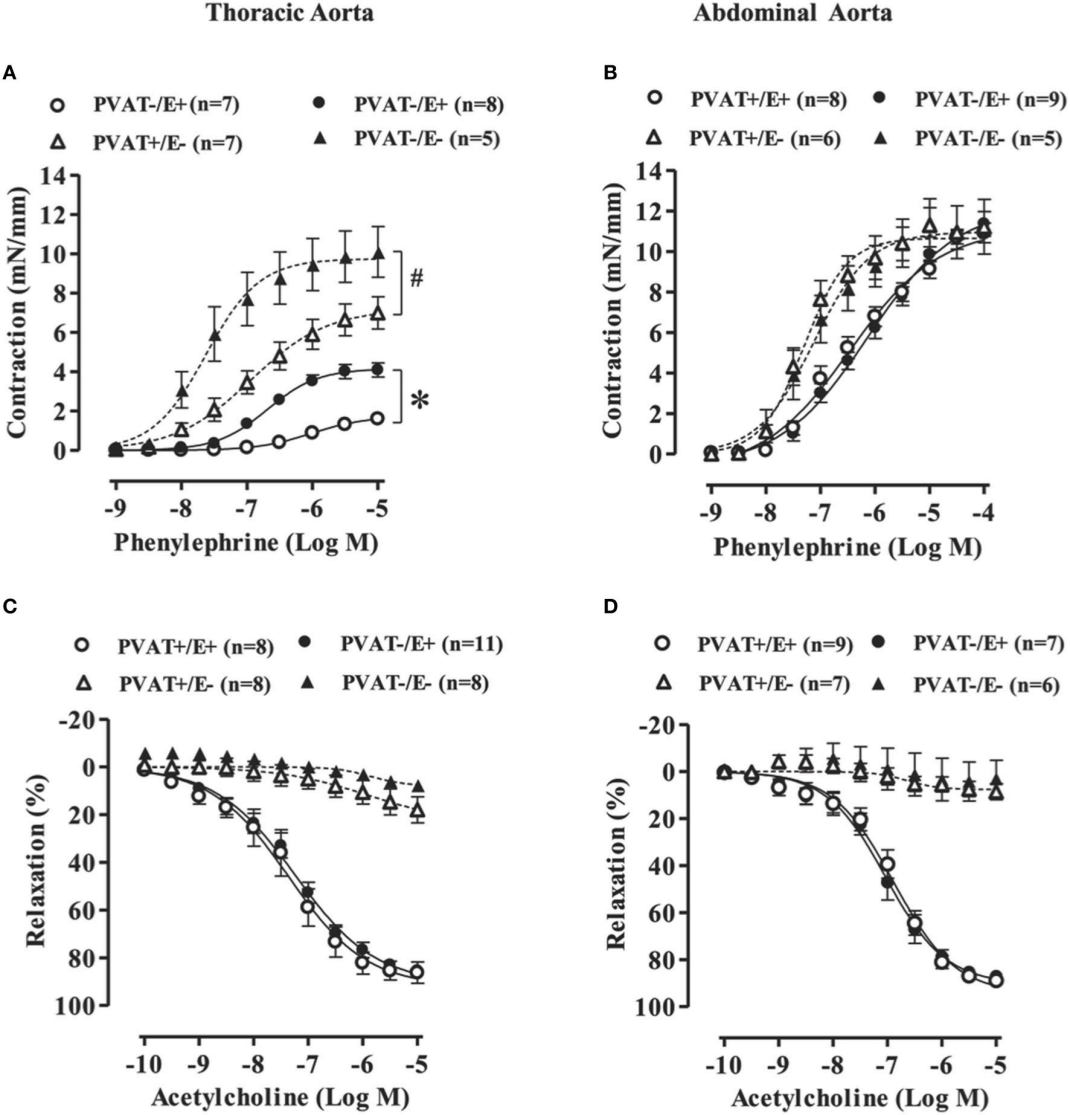
**Loss of the anti-contractile effect of perivascular adipose tissue (PVAT) in the abdominal aorta with or without endothelium**. Concentration-response curves to phenylephrine **(A,B)** and acetylcholine **(C,D)** in rat thoracic (left panel) and abdominal (right panel) aorta with (+) or without (−) endothelium (E) and PVAT. Data are expressed as the means ± SEM; the number of animals (n) is indicated in parentheses. Two-way ANOVA, *P* < 0.05: ^*^PVAT+/E+ vs. PVAT−/E+; ^#^PVAT+/E− vs. PVAT−/E−.

To determine the anti-contractile effects of PVAT in thoracic and abdominal aortic tissues, we performed concentration-response curves to phenylephrine in rings with (open symbols) or without (filled symbols) PVAT in intact (circle) or denuded (triangle) endothelium. Thoracic aortic rings with PVAT and intact endothelium (PVAT+E+) presented a significant reduction in potency and maximal response to phenylephrine when compared to rings without PVAT (PVAT−E+; Figure 1A and Table 1). Although the endothelium damage increased the phenylephrine-induced contraction (compare PVAT−/E+ vs. PVAT−/E−, Figure 1A), the anti-contractile effect of PVAT was still observed in endothelium-denuded rings. Thus, the presence of PVAT (PVAT+E−) in endothelium-denuded rings also reduced both the potency and maximal response to phenylephrine when compared to rings without PVAT and endothelium (PVAT−E−; Figure 1A and Table 1). In contrast, the presence of PVAT did not alter the phenylephrine-induced contraction in either intact or denuded endothelium abdominal aortic rings (Figure 1B and Table 1).

**Table 1:** **Potency (−LogEC50) and maximal response (Rmax) values to phenylephrine-induced contraction in thoracic and abdominal aortas with (+) or without (−) endothelium (E) and perivascular adipose tissue (PVAT)**.

	**Thoracic aorta**		**Abdominal aorta**	
	**Rmax (mN/mm)**	**−LogEC50**	**Rmax (mN/mm)**	**−LogEC50**
PVAT−E+	4.1 ± 0.3 (8)	6.6 ± 0.04 (8)	10.5 ± 1.2 (9)	6.3 ± 0.50 (9)
PVAT+E+	1.5 ± 0.1^*^ (7)	5.9 ± 0.09^*^ (7)	9.4 ± 1.5 (8)	6.6 ± 0.37 (8)
PVAT−E−	10.1 ± 1.2 (5)	7.5 ± 0.15 (5)	11.8 ± 0.9 (5)	7.0 ± 0.63 (5)
PVAT+E−	7.0 ± 0.8^#^ (7)	6.9 ± 0.12^#^ (7)	11.6 ± 3.0 (6)	7.2 ± 0.22 (6)

*Data are expressed as the means ± SEM; the number of animals is indicated in the parenthesis. Student's t-test, P < 0.05*:

* *PVAT+/E+ vs. PVAT−/E+*;

# *PVAT+/E− vs. PVAT−/E−*.

KCl-induced contractions were similar in both thoracic and abdominal aortic segments without (PVAT−E+; THO: 8.0 ± 0.5 vs. ABD: 8.7 ± 0.4 mN/mm) or with PVAT (PVAT+E+; THO: 9.4 ± 0.5 vs. ABD: 7.7 ± 0.5 mN/mm).

We also assessed the endothelium-dependent relaxation response to acetylcholine in thoracic and abdominal aorta. As expected, endothelium damage blocked the vasodilatation induced by acetylcholine in both thoracic and abdominal aorta (Figures 1C,D). However, no effects of PVAT on the acetylcholine-induced relaxation were observed in either thoracic or abdominal aorta (Figures 1C,D).

### eNOS expression and NO availability is impaired in PVAT of the abdominal aorta

**Figure 2:**
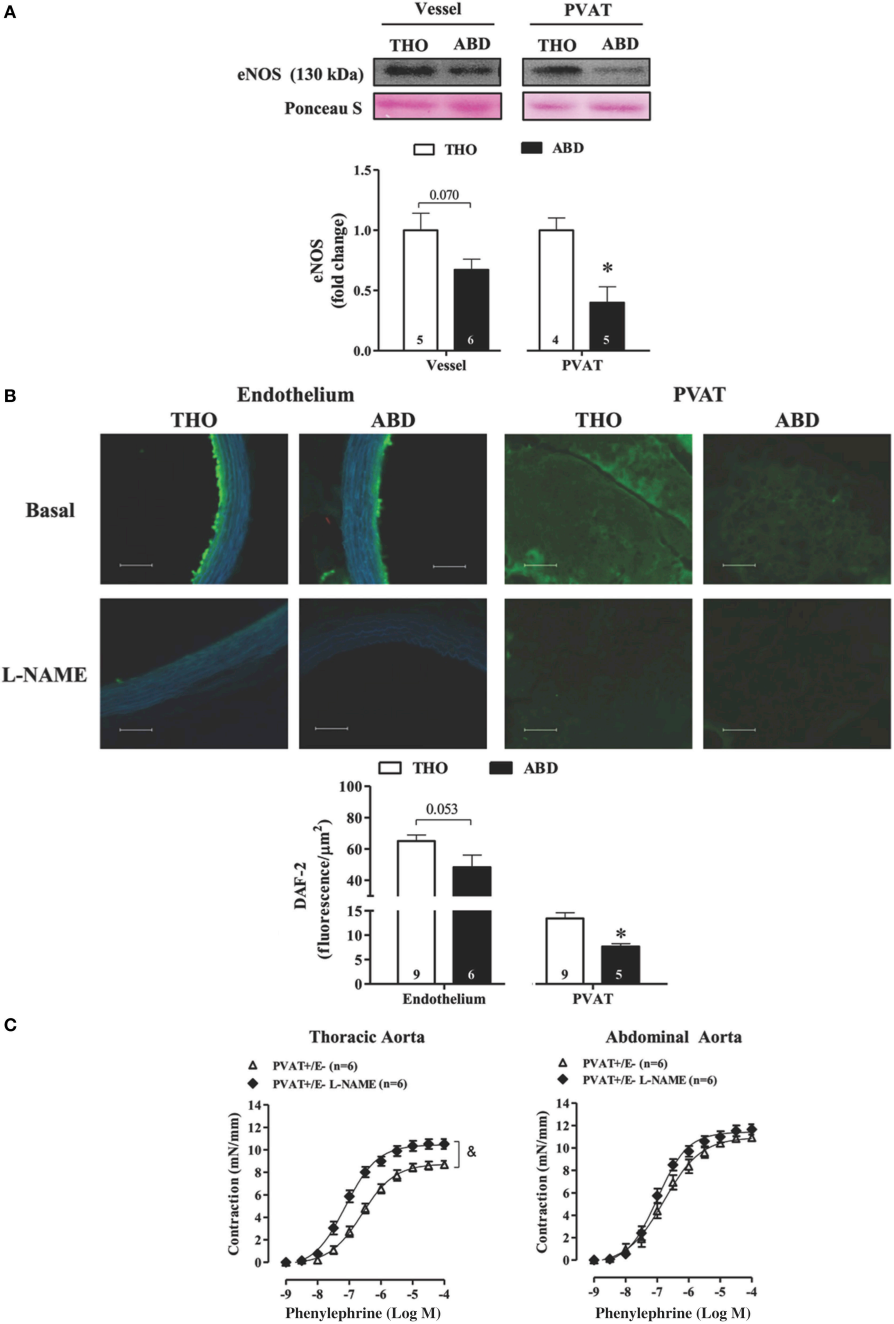
**Reduced eNOS expression and NO availability in abdominal vs. thoracic perivascular adipose tissue (PVAT)**. **(A)** eNOS protein expression in vessel walls and PVAT from thoracic (THO) and abdominal (ABD) aorta. Representative blots and Ponceau S staining were demonstrated at the upper panels of the figure and densitometric analysis is expressed as a fold change of THO expression at the bottom panel. **(B)** Top panels-Representative fluorographs of 4,5-diaminofluorescein diacetate (DAF-2) signal obtained in transverse sections of vessel walls and PVAT from THO and ABD aortic tissues in the absence (upper panel) and presence (lower panel) of L-NAME. Scale bar = 100 μm (20X objective). Bottom panel-Quantified NO availability, measured as DAF-2 fluorescence intensity in the endothelial layer and PVAT of THO and ABD aorta. **(C)** Concentration-response curves to phenylephrine in rat thoracic (left panel) and abdominal (right panel) aorta without (−) endothelium (E) and with (+) PVAT in the absence or presence of L-NAME. Data are expressed as the means ± SEM; the number of animals is indicated in the bars or in parenthesis. Student's *t*-test, *P* < 0.05: ^*^ABD vs. THO PVAT; Two-way ANOVA, *P* < 0.05: & PVAT+E− vs. PVAT+E− L-NAME.

There was a non-significant trend (*p* < 0.07) toward reduced eNOS protein expression in the abdominal when compared to the thoracic aorta (Figure 2A), whereas abdominal PVAT showed a 60% reduction in eNOS expression compared with thoracic PVAT (Figure 2A). In accordance with these data, NO availability evaluated based on DAF-2 fluorescence was not significantly altered in the endothelium of abdominal vs. thoracic aorta (*p* < 0.053), but abdominal PVAT showed a decrease in NO availability of 34% compared with thoracic PVAT (Figure 2B). L-NAME incubation significantly reduced NO bioavailability in both the endothelium and PVAT of abdominal and thoracic portions of aorta (Figure 2B).

The role of NO-derived from PVAT on phenylephrine-induced contraction was evaluated by the L-NAME incubation in thoracic and abdominal PVAT+E− rings. L-NAME increased the potency and maximal response to phenylephrine in thoracic (Rmax: PVAT+/E− = 8.7 ± 0.3 vs. PVAT+/E− L-NAME = 10.5 ± 0.4 mN/mm, *p* < 0.05; −LogEC50: PVAT+/E− = 6.6 ± 0.12 vs. PVAT+/E− L-NAME = 7.0 ± 0.07, *p* < 0.05) but not in abdominal aorta (Rmax: PVAT+/E− = 10.9 ± 0.1 vs. PVAT+/E− L-NAME = 11.6 ± 0.4 mN/mm, *p* > 0.05; −LogEC50: PVAT+/E− = 6.7 ± 0.15 vs. PVAT+/E− L-NAME = 6.9 ± 0.08, *p* > 0.05; Figure 2C).

### ROS production and lipid peroxidation did not vary along the aorta

**Figure 3:**
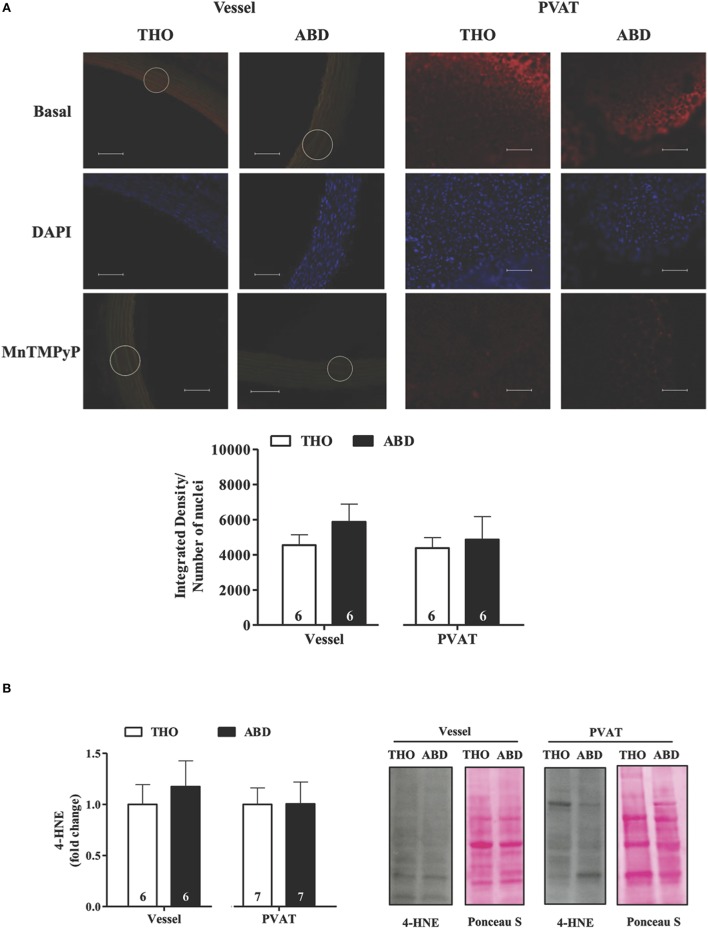
**Reactive oxygen species generation and lipid peroxidation are similar along the aorta**. **(A)** Representative fluorographs (top) and quantified (bottom) DHE fluorescence obtained in transverse sections of vessel walls and PVAT from thoracic (THO) and abdominal (ABD) aorta. DHE fluorescence was evaluated at the basal level and in the presence of the SOD mimetic MnTMPyP. Scale bar = 100 μm (20X objective). Values of the integrated density of hydroethidine-positive (EB-positive) nuclei fluorescence were normalized to nuclei number, which was analyzed by DAPI staining in each sample. **(B)** Expression of 4-hydroxynonenal (4-HNE) adducts in vessel walls and PVAT from ABD and THO aorta. Representative blots and Ponceau S staining were demonstrated at the right panel and densitometric analysis is expressed as the fold change of THO expression (left panel). Data are expressed as the means ± SEM; the number of animals is indicated in the bars of the graph. Student's *t*-test.

ROS production was detected based on DHE fluorescence (Figure 3A), and lipid peroxidation was evaluated based on the expression of 4-HNE adducts (Figure 3B). ROS production was almost fully inhibited by MnTMPyP in abdominal and thoracic aorta, suggesting superoxide as the main ROS evaluated *in situ* by DHE fluorescence in both the vascular wall and PVAT (Figure 3A). Both ROS production and lipid peroxidation were similar in abdominal and thoracic aortic tissues and PVAT (Figures 3A,B).

### Anti-oxidative profiles of abdominal and thoracic aortic tissue and PVAT

**Figure 4:**
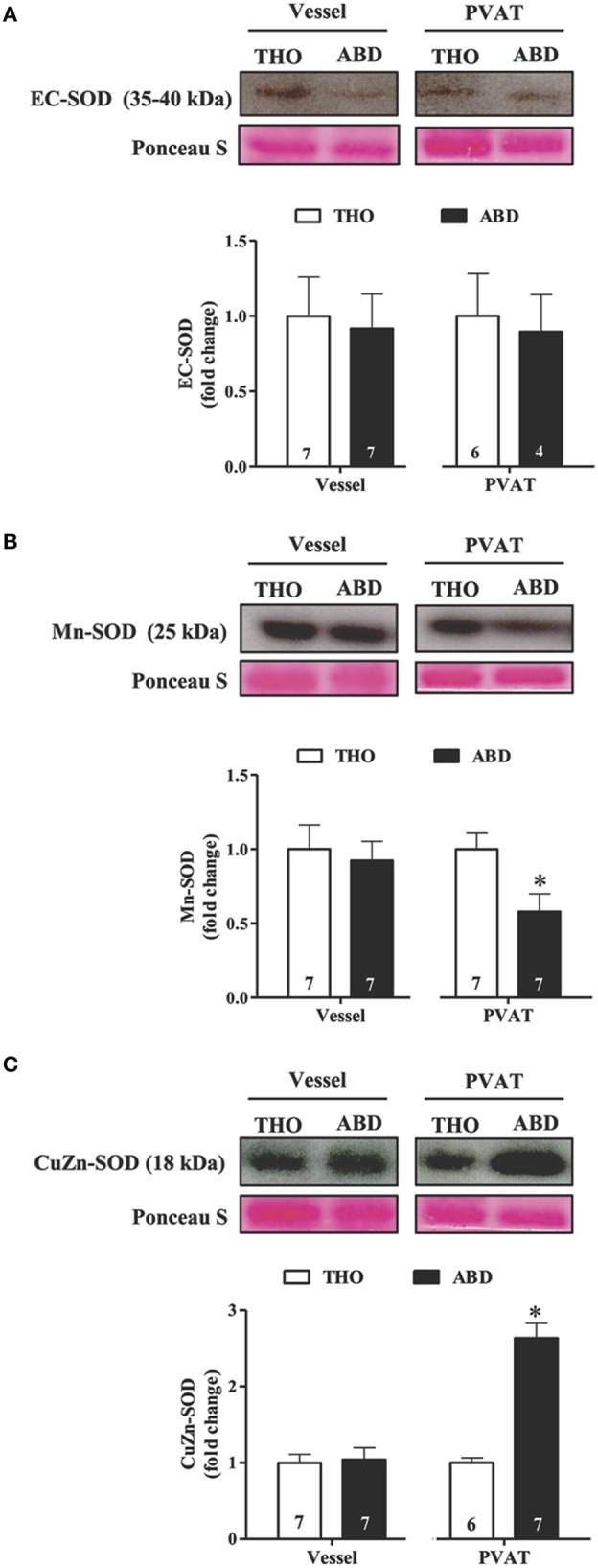
**Comparative protein expression levels of superoxide dismutase (SOD) isoenzymes in thoracic (THO) and abdominal (ABD) aortic tissues**. Protein expression of extracellular (EC)-SOD **(A)**, Mn-SOD **(B)**, and cytoplasmatic CuZn-SOD **(C)** in vessel walls and perivascular adipose tissue (PVAT) from THO and ABD aorta. Representative blots and Ponceau S staining were demonstrated at the upper panels of the figures and densitometric analysis is expressed as the fold change of THO expression at the bottom panel. Data are expressed as the means ± SEM; the number of animals is indicated in the bars of the graph. Student's *t*-test, *P* < 0.05: ^*^ABD vs. THO PVAT.

The protein expression levels of SOD isoforms were investigated in abdominal and thoracic tissues. EC-SOD did not differ between abdominal and thoracic aortic tissues and PVAT (Figure 4A), while reduced Mn-SOD expression was detected in abdominal compared with thoracic PVAT, without changes in the vascular wall (Figure 4B). In contrast, CuZn-SOD expression was increased in abdominal PVAT compared to thoracic PVAT, with no regional differences in the aortic wall (Figure 4C).

## Discussion

The present results showed that: (1) the anti-contractile effect of PVAT on alpha-adrenergic-induced contraction observed in thoracic aorta is completely lost in the abdominal section, (2) there is a significant reduction in eNOS-derived NO production in PVAT but not in the endothelium of the abdominal vs. the thoracic aorta, and (3) ROS production and lipid peroxidation levels appear to be similar along the aorta, although the relative expression levels of SOD isoforms are different. Therefore, a minor contribution of NO in the abdominal vs. the thoracic adipose depot, rather than changes in redox status, might be involved in the functional regional differences along the aorta.

Anti-contractile effects of murine thoracic periaortic fat have been demonstrated in response to several contractile agonists, including phenylephrine, serotonin and angiotensin II (Löhn et al., 2002; Fésus et al., 2007; Gao et al., 2007; Lee et al., 2011; Sun et al., 2013; Araujo et al., 2015). Importantly, the endothelium contributes to the anti-contractile effect of PVAT in the response to angiotensin II (Gálvez-Prieto et al., 2012), while it only attenuates or does not alter its effect on the alpha-adrenergic mediated contraction in the thoracic aorta (Gao et al., 2007; Lee et al., 2009, 2011). In agreement with these data, both endothelium and PVAT, independently of each other, restrain the contractile response to phenylephrine in the thoracic aorta. In contrast, in the abdominal aorta, only the endothelium exerted an anti-contractile effect in the phenylephrine-induced contraction. These data are consistent with previous data demonstrating anti-contractile effects of endothelium-derived NO in both thoracic and abdominal aortic tissues (Kleinbongard et al., 2013), while the anti-contractile function of periaortic fat on the angiotensin II-induced contraction is impaired in the abdominal section (Watts et al., 2011).

Although the contractile response to phenylephrine varies along the aorta, endothelium-dependent relaxation levels induced by acetylcholine were similar in abdominal and thoracic aorta. Similar relaxation responses to acetylcholine were also observed in abdominal vs. thoracic sections of aorta in male Sprague-Dawley rats (Oloyo et al., 2012). In addition, PVAT did not play a role in the aortic relaxation response to acetylcholine, as previously demonstrated (Ketonen et al., 2010; Gálvez-Prieto et al., 2012).

NO availability using DAF-2 fluorescence was previously demonstrated in PVAT of mouse mesenteric arteries (Gil-Ortega et al., 2010) and thoracic aorta (Xia et al., 2016). Here, for the first time, we demonstrated that NO availability in PVAT was significantly reduced in abdominal vs. thoracic aorta, whereas endothelium-derived NO levels were similar between the two portions of the aorta. The reduction in DAF-2 fluorescence in the abdominal periaortic fat was accompanied by a similar magnitude of reduction in eNOS expression in this tissue, while eNOS expression in the endothelium remained the same along the aorta. Reinforcing these results, it is interesting to observe that in PVAT preserved but endothelium denuded abdominal aortic rings the inhibition of NO synthesis did not change the phenylephrine-induced contraction, while it was increased in thoracic aortic rings. The cell-specific localization of the higher eNOS protein expression in thoracic aortic PVAT remains to be elucidated. Because microvessels are present throughout the thoracic PVAT, we cannot rule out endothelial cells from these vessels as a source of NO in PVAT, albeit the vast majority of eNOS positive cells in PVAT are adipocytes (Xia et al., 2016).

It is known that the abdominal aorta exhibits a different PVAT phenotype compared to the thoracic aorta: while abdominal PVAT exhibits a phenotype of pro-inflammatory fat, thoracic periaortic fat shares characteristics of BAT (Padilla et al., 2013). Interestingly, enlargement of lipid droplet morphology has been observed in thoracic periaortic fat from obese mice (Fitzgibbons et al., 2011). This change in phenotype induced by a high-fat diet is accompanied by a significant reduction in PVAT-derived NO production related to decreased eNOS phosphorylation (Xia et al., 2016). However, aerobic exercise training can overbrowning and enhances the eNOS expression in thoracic PVAT (Araujo et al., 2015). These studies support the hypothesis that physiological white fat depots surrounding the abdominal aorta may not exhibit a significant anti-contractile function due to less pronounced eNOS expression and NO production and/availability.

In the absence of PVAT and endothelium, abdominal and thoracic portions of the aorta exhibit similar raw forces in response to phenylephrine, suggesting both as major mechanisms involved in the regional contractile differences along the aorta. However, we cannot exclude other intrinsic factors. Although contraction in response to phenylephrine of rat abdominal and thoracic aorta is mediated via alpha1D-adrenergic receptor subtype (Asbún-Bojalil et al., 2002), prostanoids derived from smooth muscle cells contribute to contraction only in the abdominal aorta (Lamb et al., 1994). In addition, distal aortic segments are stiffer than proximal ones (Sokolis et al., 2008; Devos et al., 2015), although the mechanisms underlying this regional difference within the aorta is not fully established. Previous study has shown that impairment of eNOS activity is an important mechanism inducing vascular extracellular matrix remodeling in a model of abdominal aortic aneurism (Gao et al., 2012). Therefore, the substantial reduction in NO availability observed in the present study at the abdominal aorta might be a relevant factor implicated in the susceptibility of this part of the aorta to vascular injury.

It is well-known that NO availability is impaired by the spontaneous reaction of NO with superoxide anion causing the generation of peroxynitrite, a potent oxidant with potential cytotoxicity. Gil-Ortega et al. (2014) demonstrated that an enhanced superoxide generation in PVAT surrounding mesenteric arteries of obese mice is associated with a loss of the anti-contractile effect of PVAT on the contractile response of mesenteric arteries to noradrenaline. Therefore, we attempted to investigate whether oxidative differences along the aorta could be involved in the impaired anti-contractile function and NO availability noted in the abdominal aorta. Interestingly, no regional differences were noted in either superoxide generation, based on DHE fluorescence, or lipid peroxidation, assessed based on the presence of 4-HNE-protein adducts between the vascular wall and PVAT of abdominal and thoracic portions of the aorta. Early dysfunction of thoracic aortic PVAT was associated with increased lipid peroxidation, evaluated based on the presence of thiobarbituric acid reactive species (TBARS), in response to high fructose diet (Rebolledo et al., 2010). However, this change reflected those reported in abdominal adipose depots (Alzamendi et al., 2009), suggesting that they are the consequence of general rather than local oxidative stress involved in the PVAT control of vascular function.

The major enzymatic control of superoxide anion levels in the vessel wall is exerted by SOD isoforms (Faraci and Didion, 2004). SOD activity was also detected in PVAT of murine mesenteric arteries and thoracic aorta, playing an important role in the redox status of vascular wall (Rebolledo et al., 2010; Gil-Ortega et al., 2014). SODs convert superoxide anion into hydrogen peroxide, thereby protecting NO availability and signaling. Although the three isoforms of SOD catalyze the same reaction, they differ in both localization and their importance for vascular function. Here, we noted similar patterns of SOD expression between the abdominal and thoracic aortic walls. However, in comparison with the thoracic PVAT, abdominal PVAT exhibited reduced protein levels of Mn-SOD and enhanced cytosolic CuZn-SOD. Interestingly, CuZn-SOD overexpression protects the aorta from lipid peroxidation and DNA fragmentation, whereas Mn-SOD heterozygous-deficient mice exhibited enhanced aortic lipid peroxidation and apoptosis, mechanisms of importance for the development of atherosclerosis (Guo et al., 2001). However, the functional significance of differences in vascular expression or activity in PVAT is still poorly understood; future studies will be necessary to address this question.

Taken together, the present results demonstrate that, compared with the thoracic aorta, the anti-contractile function of PVAT is impaired in the abdominal portion of the aorta through a reduction in eNOS-derived NO production without changes in ROS generation and lipid peroxidation. Knowing that aortic atherosclerotic lesions and aneurysms are predominant in abdominal rather than thoracic portions of the aorta, these findings highlight a new specific regional role of PVAT depots in the control of vascular function that may drive differences in susceptibility to vascular injury between these two portions of the aorta.

## Author contributions

Conceived and designed the study: LR, AD. Performed the experiments: JV, MF. Analyzed and interpreted the data: JV, MF, LR, AD. Wrote the manuscript: JV, MF, LR, AD.

## Funding

This work was supported by Fundação de Amparo à Pesquisa do Estado de São Paulo (FAPESP grants 14/07947-6 and 14/20303-0) and Conselho Nacional de Desenvolvimento Científico e Tecnológico (CNPq grant #447507/2014-1). AD and LR are research fellows from Conselho Nacional de Desenvolvimento Científico e Tecnológico (CNPq, Brazil).

### Conflict of interest statement

The authors declare that the research was conducted in the absence of any commercial or financial relationships that could be construed as a potential conflict of interest.
